# A Few Observations upon the Subject of Digestion

**Published:** 1867-08

**Authors:** R. Dexter

**Affiliations:** Chicago, Ill.


ARTICLE XXXIV.

### A FEW OBSERVATIONS UPON THE SUBJECT OF DIGESTION.

By R. DEXTER, M.D., Chicago, Ill.

*Food—Classifications of.*

| | | | |
|---|---|---|---|
| Nitrogenous | Albumen, Caseine & Fibrine. | Non-Nitrogenous. | Hydro Carbons and Fats. CARBO HYDRATES. Sugar, Starch, and Gums. |

*Food—Physiological Classification of.*

| | |
|---|---|
| Tissue-making Food. | Albumen, Fibrine, and Caseine. |
| Sugar-making Food. | Gluten, and Chondrine. |
| Fat-making Food. | Sugar, Starch, and Fats. |

*Processes Wrought in Different Organs.*

| | |
|---|---|
| Mouth—Changes in. | Mastication, and Insalivation. We have partial conversion of Starch into Glucose. |
| Stomach — Changes in. | Albumen, Fibrine, Caseine, and Animal Jelly. |
| Duodenum—Changes in. | Sugar, Starch, and Fats. |

As the result of the digestive processes, we have the following:
Albuminose, consisting of Albumen, Fibrine, and Caseine.
Gelatinose, consisting of Gluten, and Chondrine.
Glucose, consisting of Sugar, and Starch.

Adipose, consisting of Fats.

The whole digestive mass gains entrance into the blood by two separate routes, viz.: lymphatics and veins.

1. Lymphatics of the small intestines absorb the adipose emulsion with an albuminous coating of each globule.

2. The balance of the albuminose, with the whole of the gelatinose, and gleucose are absorbed by the veins of the small intestines, and pass to the liver through the portal vein.

3. In the liver the albuminose becomes albumen, the gelatinose becomes sugar, and the glucose becomes fat.

4. Liver sugar is carried to the lungs, where it is converted into lactic acid.

5. In the transit of the emulsion through the intestinal lymphatics fibrine and the white corpuscles of the blood are organized and elaborated.

6. In the spleen a portion of fibrine white corpuscles and nurine of the blood are organized and elaborated.

7. (Conjectural.) Old red corpuscles disorganize in the spleen, and from the debris fibrine is the only constituent that serves any useful purpose; the residue is excreted.

8. The white corpuscles, in their passage through the spleen, elongate and burst, setting their nuclei free. These nuclei absorb hæmatin, and become red corpuscles, while the wall of the bursted corpuscles fibrius.

9. But the liver is the grand laboratory of red corpuscles. The hepatic vein is richer in both red and white corpuscles than any other vessel of the body. The splenic vein is next in richness of red and white corpuscles to the hepatic.

10. Assimilation takes place outside of the blood vessels. The exuded plasma saturates every structure of which we are composed, and decayed portion of the tissues are replaced by the appropriation of a sufficient amount of the nutritive pabulum.

11. The bile is not a digestive fluid; it is both secretory and excretory. A secretion always serves some further useful purpose in the system, and is never found in the blood.

12. There are no essential changes wrought upon animal fats either by digestion or assimilation.

